# Herpesviruses that Infect Fish

**DOI:** 10.3390/v3112160

**Published:** 2011-11-08

**Authors:** Larry Hanson, Arnon Dishon, Moshe Kotler

**Affiliations:** 1 Department of Basic Sciences, College of Veterinary Medicine, Mississippi State University, P.O. Box 6100, Starkville, MS 39759, USA; 2 KoVax Ltd., P.O. Box 45212, Bynet Build., Har Hotzvim Inds. Pk., Jerusalem 97444, Israel; E-Mail: Arnon.Dishon@kovax.co.il; 3 Department of Pathology, Hadassah Medical School, the Hebrew University, Jerusalem 91120, Israel; E-Mail: moshek@ekmd.huji.ac.il; 4 The Lautenberg Center for General and Tumor Immunology, Hadassah Medical School, the Hebrew University, Jerusalem 91120, Israel

**Keywords:** alloherpesvirus, herpesvirus latency, Koi herpesvirus, *Cyprinid herpesvirus* 3, channel catfish virus, *Ictalurid herpesvirus* 1

## Abstract

Herpesviruses are host specific pathogens that are widespread among vertebrates. Genome sequence data demonstrate that most herpesviruses of fish and amphibians are grouped together (family *Alloherpesviridae*) and are distantly related to herpesviruses of reptiles, birds and mammals (family *Herpesviridae*). Yet, many of the biological processes of members of the order *Herpesvirales* are similar. Among the conserved characteristics are the virion structure, replication process, the ability to establish long term latency and the manipulation of the host immune response. Many of the similar processes may be due to convergent evolution. This overview of identified herpesviruses of fish discusses the diseases that alloherpesviruses cause, the biology of these viruses and the host-pathogen interactions. Much of our knowledge on the biology of *Alloherpesvirdae* is derived from research with two species: *Ictalurid herpesvirus* 1 (channel catfish virus) and *Cyprinid herpesvirus* 3 (koi herpesvirus).

## 1. Introduction

Herpesviruses are important pathogens in fish. They are wide spread among mammals, birds and fish, and most are thought to have evolved in the same host species over long periods. They have large genomes and intricate mechanisms to persist in the host. This precise specialized interaction has resulted in a high level of host specificity and even the evolution of distinct species within the same host. Most herpesviruses infections are unapparent or cause mild disease in natural conditions, but in an immune compromised host, aberrant host or in an environment that promotes transfer of high doses of virus to a naïve host, these viruses can be highly pathogenic. Over 14 known herpesviruses are associated with disease outbreaks in fish (Table 1). However, host and tissue specificity of many herpesviruses make them recalcitrant to cell culture. Thus, there are many more disease causing herpesviruses that have yet to be characterized. Table 2 is a listing of suspected herpesviruses seen by electron microscopy but not cultured and not yet confirmed by molecular methods. It is interesting to note that of the identified herpesviruses only one has been found in Chondrichthyes (sharks and rays), and none have been identified in lampreys or hagfish. This may be simply because these fish species are not as important for food fish, culture and ornamental use as are the bonyfish. Herpesvirus caused diseases of fish (namely carp pox, caused by Cyprinid herpesvirus 1) have been recognized for centuries. Devastating diseases in aquaculture caused by herpesviruses include, channel catfish virus disease, *Oncorhynchus masou* virus disease and koi herpesvirus disease (see [1] for more information about specific diseases). Because the herpesviruses of fish can cause devastating diseases and herpesviruses of mammals and birds are important pathogens in human and veterinary medicine, several fish herpesviruses have been well characterized and select fish herpesviruses have been the subjects of comparative studies.

## 2. Molecular Characteristics of Fish Herpesviruses

The genomes of over 48 different herpesviruses have been sequenced, including 3 fish viruses [2–4], two frog viruses [5] and an oyster virus [6]. This has allowed the whole genome sequence comparisons and detailed phylogenetic analyses. The findings of these studies revealed that the order *Herpesvirales* is composed of three genetically distinct groups of viruses. Because of the genetic distance between the groups, they have been classified into 3 separate families: *Herpesviridae*-predominantly pathogens of mammals, birds and reptiles, *Alloherpesviridae*-predominantly pathogens of fish and amphibians and *Malacoherpesviridae*- which was identified in a mollusk (oyster) [7,8]. Among these, *Herpesviridae* has been classified in to three subfamilies; *Alphaherpesvirinae*, *Betaherpesvirinae* and *Gammaherpesvirinae* [8]. There is almost no sequence similarity between the families and, in fact, in a large comparison of all large DNA viruses based on amino acid sequences of predicted genes Wu *et al.* [9] found that the three families of *Herpesvirales* do not cluster to form a monophyletic group.

### 2.1. Gene Sequence Conservation

One protein among the many shows some sequence conservation among the *Herpesvirales*. This is the ATPase subunit of the terminase, a protein involved in packaging genome into the capsid during virion assembly [6,7,56]. The encoding gene also appears to be related to a similar gene in T4 like bacteriophages. This and structure similarities of capsid subunits suggest a prokaryotic origin of *Herpesvirales* [7,8]. Within *Alloherpesviridae* that have been sequenced there are 12 genes that are consistently conserved [4]. Seven of these genes encode proteins involved basic structure or essential functions in replication such as capsid morphogenesis (capsid triplex protein 2, capsid protease and scaffolding protein, and the major capsid protein), DNA replication (DNA helicase, DNA polymerase, and primase) and DNA packaging (ATPase subunit of the terminase). The other five conserved genes encode proteins with unknown functions. Conserved regions with these identified conserved proteins have been used to establish degenerate primers that allow PCR based targeted gene sequence amplification and for sequence comparisons [10,57]. This method was used by Waltzek *et al.* [10] to amplify and sequence a portion of the DNA polymerase gene, ATPase subunit of the terminase gene and allow phylogenic assessment of 13 fish and amphibian herpesviruses. This study showed that there were 2 monophyletic clades within *Alloherpesviridae* with AngHV1, CyHV1, CyHV2, and CyHV3 in Clade 1 and IcHV1, IcHV2, AciHV1, AciHV2, SalHV1, SalHV2, SalHV3, RaHV1 and RaHV2 in Clade 2 (see Table 1) [10]. Furthermore, it demonstrated the utility of degenerate PCR for characterizing unculturable herpesviruses (SalHV3). This method was also applied to characterize an unculturable herpesvirus of Atlantic cod and the placement of it in Clade 2 [30]. Subsequently the evaluation of an 8 kb gene block between DNA polymerase gene and the ATPase subunit of the terminase gene in IcHV2 and AciHV2 confirmed the 2 clade designation; also the new sequence data allowed Pilchard HV to be tentatively placed within Clade 2 [34]. Additionally the phylogenic studies were facilitated by partial genome sequencing of SalHV1 [58] and recently AciHV2 [40]. The new AciHV2 data provided enough evidence that Clade 2 could be subdivided into 2 distinct clades, one containing the frog herpesviruses and the other containing the fish herpesviruses of Clade 2. They proposed that the 3 clades be designated as subfamilies [40]. There is one interesting outlier among fish herpesviruses. Sequence analysis of small fragment of the DNA polymerase gene of tilapia HV suggests that it may be a member of *Herpesviridae* [38]. In another report DNA from the fibropapilloma-associated turtle herpesvirus, a member of *Alphaherpesvirinae*, was found in various tissues of cleaner wrasses. However it is not known if the virus was infecting the fish [59].

### 2.2. Genome Structure

**Figure 1.**
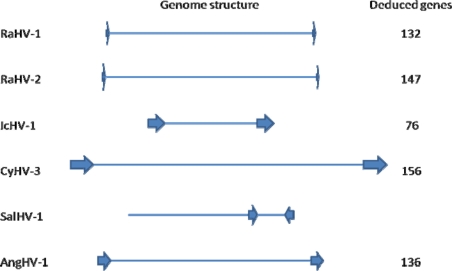
A schematic of the genome structure and coding capacity of characterized alloherpesviruses.

Genome sequencing has revealed the structural characteristics of the Alloherpesviruses. Of the six structurally evaluated genomes, all are packaged as unit length, linear genomes ranging in size from 134 kb (IcHV1 one of the smaller herpesviruses), to the largest known herpesvirus genome, 295 kb (CyHV3) (Figure 1). Five are arranged with terminal direct repeat sequences. The terminal repeats range from in size from 636 bp in RaHV2 [5] to 22 kb in CyHV3 [3]. The exception is SalHV1 [58]. Its 174.4 kb genome is composed of a 133.4 kb unique long sequence, and a 25.6 Kb unique short fragment flanked by 7.7 kb inverted repeats. This structure allows two isomeric orientations of the unique short fragment similar to the genome structure of the varicella-zoster virus (VZV) a mammalian alphaherpesvirus [60].

In addition to the short direct repeats, the ranid herpesviruses contain large regions of internal repeats (one in RaHV1, 153 bp elements, and five in RaHV2, 133–175 bp elements) within their genome as seen in the Epstein-Bar virus (EBV) a mammalian gammaherpesvirus [5,60]. Also, the ranid herpesviruses are unique among sequenced herpesviruses in that they encode a DNA (cytosine-5-)-methyltransferase and have extensively methylated genomes similar to iridoviruses.

In general, the gene structures of *Alloherpesviridae* are simple with 2–3 genes of the whole genome being spliced. Often tandem genes share the termination/polyadenylation sequences, so overlapping transcription may occur, and it is generally thought that the first open reading frame in the transcript is expressed since no internal ribosomal binding sites have been described. One unusual gene structure has been described for the gene encoding the ATPase subunit of terminase. Like the other alloherepsviruses, it is coded by 3 exons, but in the ranid herpesviruses the first exon is encoded on the opposite DNA strand from the second and third exons [5].

### 2.3. Virion Structure

Given the discordance in genome sequences among *Herpesvirales,* it is reassuring to note that herpesvirus virion structures are remarkably conserved. The genome is densely packed as a visible core within a 115–130 nm diameter icosahedral capsid. This capsid is composed of 162 capsomeres. The nuleocapsid is embedded in a amorphous layer called the tegument, and this assembly is surrounded by an envelope, a lipid bilayer membrane derived from a host cell membrane containing various glycoproteins [8]. Booy *et al.* [61] used cryoelectron microscopy computer-based image reconstruction to compare the structure of human herpesvirus 1 (herpes simplex virus 1) (*Herpesviridae* ) to IcHV1. Subsequently, Davison *et al.* [6] evaluated Ostreid herpesvirus 1 (OsHV1) (*Malacoherpesviridae*). They found that all three had similar capsid structure composed of 150 hexomers and 12 pentomers. The capsomers have 9 nm (IcHV1) to 11 nm (HHV1) chimney-like protrusions with an axial channel through each capsomer.

## 3. Biological Characteristics of Fish Herpesviruses

Even though alloherpesviruses are distantly related to *Herpesviridae*, there are many similarities in the way they infect, replicate and persist in the host. The three main shared characteristics are a high level of host specificity, the apparent ability to intricately interact with the host defenses and the ability to establish long-term latency. In this section we will briefly discuss common themes among alloherpesviruses, then in the subsequent two sections we will review in detail CyHV3 and IcHV1, because these are the best characterized alloherpesvirus, and they represent model species for the two identified genetically distinct clades.

### 3.1. Alloherpesviruses Display a High Level of Host Specificity

Similar to Herpesviridae all characterized alloherpesviruses to date appear to cause disease in only one species of fish or in closely related members of the same genus (*i.e.*, SalHV2) (see Table 1). This species specificity is often reflected in cell culture. For example, all three identified cyprinid herpesviruses only grow in cyprinid cell lines, ictalurid herpesvirus 1 seems restricted to catfish cell lines, salmonid herpesvirus 1 and 2 are restricted to salmonid cell lines, the acipenserid herpesviruses are restricted to sturgeon cell lines and walleye herpesvirus appears restricted to walleye cell lines. There are exceptions to this though, ictalurid herpesvirus 2, grows well in a centrarchid cell line (BF2) [21] and anguillid herpesvirus 1 grows to some extent in cyprinid (EPC, FHM) and salmonid cells (RTG-2) [11]. This host specificity partially explains that high number of fish herpesviruses that are recalcitrant to propagation in cell culture; there is no suitable cell line available from the affected species.

### 3.2. Alloherpesviruses Are Epitheliotrophic

As can be seen from the summary of diseases listed in Tables 1 and 2, most alloherpesviruses cause primary pathology to epithelial cells with these cells showing signs of virus replication. Characteristic histological changes in diseased fish with alloherpesvirus infections include epidermal cell necrosis (smooth dogfish HV, AciHV1), syncytia formation (PlHV1), epidermal cell hypertrophy (GaHV1, Pacific cod HV, pilchard HV, EsHV, PlHV1), epidermal or branchial hyperplasia (AciHV1, AciHV2, SalHV3, CyHV3, flounder HV, PeHV), hyperplasia and papillomas (CyHV1, SalHV2, golden ide HV, sheatfish HV, European smelt HV, rainbow smelt HV) and renal adenocarcinoma (RaHV2). Cells that are infected often display enlarged nuclei with marginated chromatin.

### 3.3. Alloherpesviruses Establish Latent Infections

Similar to members of Herpesviridae, the alloherpesviruses that have been evaluated appear to establish long-term latent infections. The primary indication of this is the ability to detect viral genomic DNA in survivors of a productive primary infection without being able to detect infectious viruses. Latency has been indicated in CyHV1 [62], CyHV3 [63], SalHV2 [64] and IcHV1[65].

## 4. Cyprinid Herpesvirus 3 — A Model Clade 1 Alloherpesvirus

A deadly viral carp disease characterized by severe gill necrosis was detected in the United Kingdom in 1996, but the disease was initially described by Ariav and coworkers in 1998, following the eruption of the fatal disease in several carp farms along the Israeli Mediterranean coast [66]. The disease was not restricted to the United Kingdom and Israel and shortly after, reports appeared describing a similar disease with mass mortality in countries all over the world [17,67–70].

The pathogen, originally designated koi herpesvirus, [17] was reclassified as Cyprinid herpesvirus 3 (CyHV3). Because CyHV3 has become a major economic threat to the common carp and koi rearing industries worldwide, it has been subject for many applied and basic studies. Indeed, during the last decade a large number of scientific reports and several review articles have been published describing the biological and molecular characteristics of the virus [69,71–74]. Consequently, this review will provide a short CyHV3 disease overview, and then we will summarize the methods used to reduce the threat of the virus, and discuss the issue of CyHV3 latency.

### 4.1. CyHV3 — “Disease Overview”

#### The CyHV3 disease:

Since 1998 many carp and koi farms have been afflicted by a disease with a high mortality rate, resulting in a drastic reduction in production. This disease is caused by CyHV3 [17,75–79] and has been observed in many farms, lakes, and rivers worldwide [17,79–81]. The disease appears in ponds during spring and fall, when the water temperature ranges from 18 °C to 28 °C and is lethal to 80 to 100% of the fish. Mortality occurs within 6 to 22 days post infection (d.p.i.), peaking at between 8 and 12 d.p.i. [76]. Studies performed in controlled environments confirmed that the virus induces the disease and propagates only at this permissive temperature range [17,76,82].

#### Clinical Signs:

The virus is highly contagious, spreads from infected to healthy fish sharing the same pond. Clinical signs appear three days post infection and include lethargy, gasping movements in shallow water suffering from suffocation. These signs are followed by gill necrosis coupled with increased levels of opportunistic parasites and bacteria [66], sunken eyes, pale patches on skin, and increased mucus secretion [17,69,76,78,82–84].

#### Histopathology:

**Figure 2.**
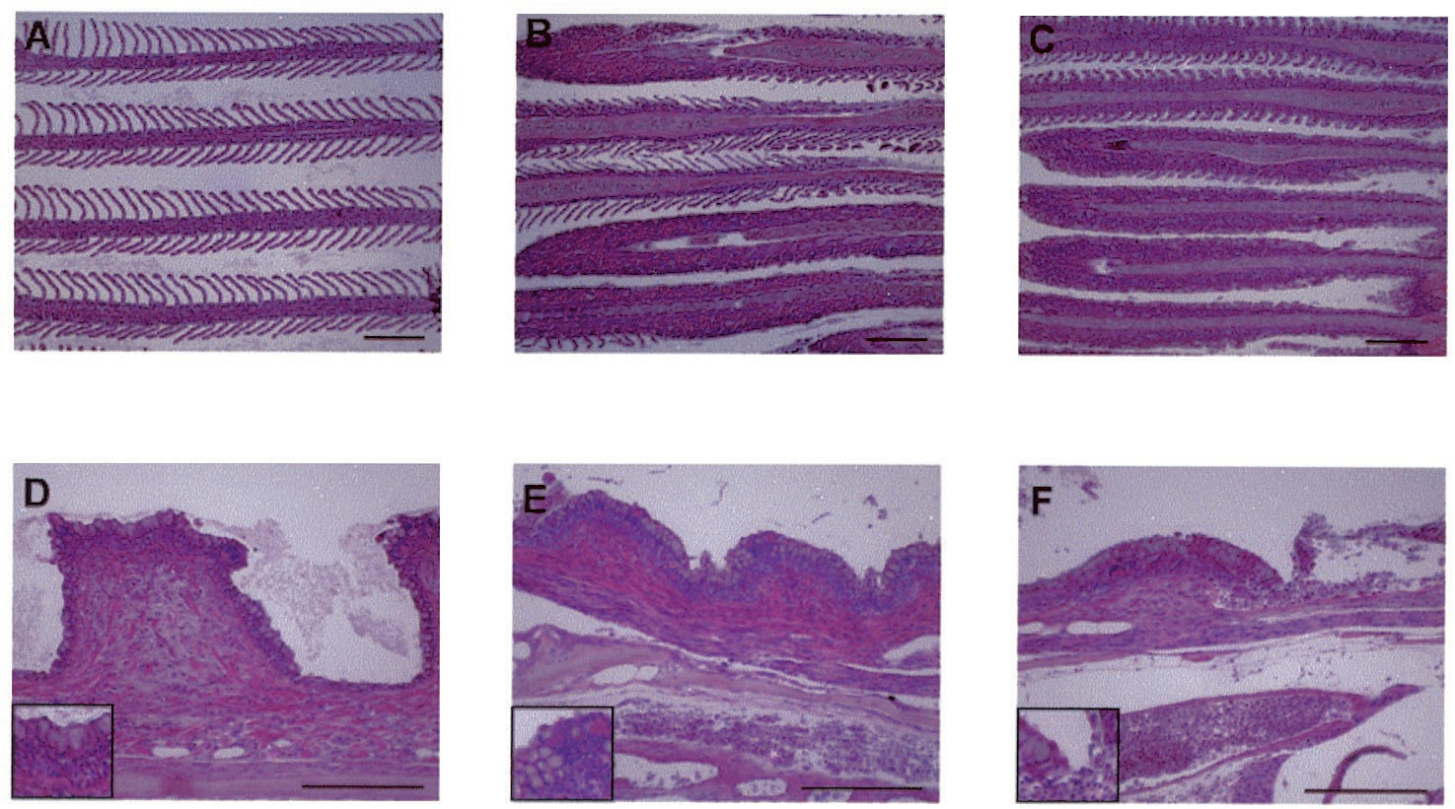
Photomicrographs of histological sections of the gills of CyHV-3 infected. (**A** to **C**) Gill filaments. (**A**) Uninfected. (**B**) 2 days post infection (p.i.) many lamellae are infiltrated by inflammatory cells. (**C**) 6 days p.i. all lamellae are heavily infiltrated. (**D** to **F**) Gill rakers. (**D**) uninfected (**E**) 2 days p.i. increased inflammatory infiltrate is present in the subepithelial zone. (**F**) 6 days p.i., the inflammatory process is more pronounced, with sloughing of the overlying epithelium (upper right). All of the sections were stained with hematoxylin and eosin. The insets in the lower left corners are of areas in the centers of the respective photomicrograph. Bars, 200 μm. Copyright © American Society for Microbiology [83].

In sick fish the most prominent lesions are observed in the gill, skin, kidney, spleen, liver and gastrointestinal systems [17,83]. Pathological changes were noted in the gills as early as 2 d.p.i., as evidenced by a loss of lamellae followed by complete effacement of the gill architecture accompanied by severe inflammation in nearly all of the filaments. The effects in the gill rakers are more prominent than the changes observed in the filaments. These include increased subepithelial inflammation and congestion of blood vessels in the gill arch, accompanied by attenuation of the length of the rakers (Figure 2). In addition to the gills, the most prominent pathological changes were noted in the kidneys. A mild peritubular inflammatory infiltrate was evident as early as 2 d.p.i. On day 6, a heavy interstitial inflammatory infiltrate was observed, along with congestion of blood vessels. When evaluated by immunohistochemistry, these interstitial cells display virus proteins by day 6 and tubule cells are positive for viral proteins by day 10. Cells Liver sample analysis showed mild inflammatory infiltrates located mainly in the parenchyma, while brain sections showed focal meningeal and parameningeal inflammation [83].

#### Virus isolation:

Virus was isolated by infection of KF-1 [17], KFC and CFC [77,83] lines with cell extracts prepared from kidneys and gills from sick fish. CyHV3 propagates well in these cell lines as well as in CCB [85] and several other cell lines, inducing severe CPE in 3–5 days post infection. The cytoplasm of infected cells becomes extremely vacuolated. These cultured cells produce virus up to 10^5^ to 10^6^ PFU/mL.

#### Host specificity:

Although the disease is highly contagious, it appears restricted to *Cyprinus carpio* (koi and common carp) populations. In experimental challenges, tilapia (*Oreochromis niloticus*), silver perch (*Bidyanus bidyanus*), silver carp (*Hypophthalmichthys molitrix*), goldfish (*Carassius auratus*) and grass carp (*Ctenopharyngodon idella*) were found to be fully resistant to CyHV3 infection, even after long cohabitation with diseased carp at the permissive temperature [76]. In contrast, PCR analysis demonstrates that goldfish can be infected with CyHV3 [86–88] and cohabitation of carrier goldfish can transfer the virus to susceptible carp [87] but the infected goldfish showed no signs of disease. Also, koi × goldfish and koi × crucian carp (*Carassius carassius*) hybrids are susceptible to CyHV-3 infection, develop CyHV-3 disease and suffer high losses [89].

Resistance of fish to CyHV3 infection could be due to lack of specific virus receptors, innate cellular immunity, or because of the host’s intensive immune response against the virus. Determining the susceptibility of cultured cells derived from cyprinid and non-cyprinid species to CyHV3 indicate that resistance of fish to CyHV3 is not solely determined at the cell level, and cells derived from cyprinid species manifest a differential resistance to virus propagation [18].

#### Dissemination of the Virus:

The rapid spread of this disease is probably due to the intensive worldwide trade of these splendid fish, mostly without veterinary supervision. Molecular studies demonstrate more genetic diversity in the European strains suggesting that the virus diverged for a longer period and likely disseminated from Europe [90,91]. This spread may have been regionally augmented by the use of natural vaccinations (as discussed later). However, it is not clear how the virus disseminates from pond to pond. One possibility is that birds or other predators transfer contaminated fish to geographically closed bodies of water. Virus harvested from tissue cultures remains infective in water for at least 4 h [76], explaining the highly contagious nature of the virus in ponds.

Based on the detection of CyHV3 in gill mucus and lamellae [83,92], it is likely that virus infects the fish via the gills, replicates there, induces mucosal sloughing and necrosis, and is then shed into the water. From the gills, the virus can be rapidly transferred to the kidneys, where it resides in leukocytes and induces severe interstitial nephritis. Localization of the virus within white blood cells raises the intriguing possibility that the virus is rapidly transferred to the viscera via infected white blood cells and then multiplies in the epithelial cells of the kidney and intestine.

The virus is released into the water either through shedding or together with the sloughed epithelial and inflammatory cells resulting from severe local inflammation. The ability to invade the fish through the gills, multiply there, and then be released through the water is analogous to the case for respiratory viruses in mammals that infect the respiratory epithelium, replicate there, and are spread through air droplets and aerosols. However, large amounts of viral DNA were found in the gut early after infection [92], and clusters of virus particles were detected by electron microscopy in the intestinal system early post infection [76], suggesting that the virus penetrates the fish body through the digestive system. By using a firefly luciferase (LUC) expression virus it was elegantly demonstrated that virus penetrates fish via the skin on the fins and body from where it rapidly disseminate to fish organs [93].

#### Morphology of purified virus and maturation in the cell

**Figure 3.**
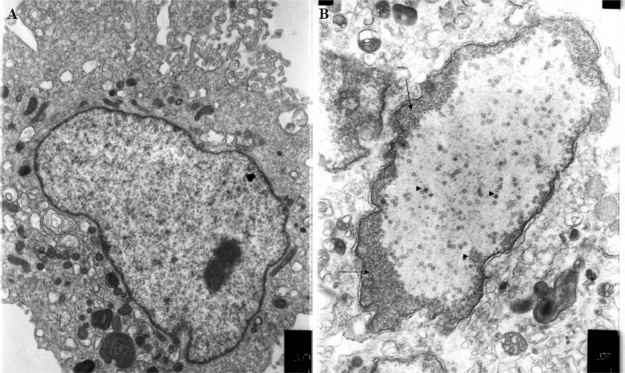
Electron micrographs of (**A**) non-infected and (**B**) CyHV-3 infected cells at 3 d.p.i. Arrows indicate margination of chromatin in the infected nucleus, arrow heads show viral capsid accumulation in the central and peripheral zones of the nucleus.

**Figure 4.**
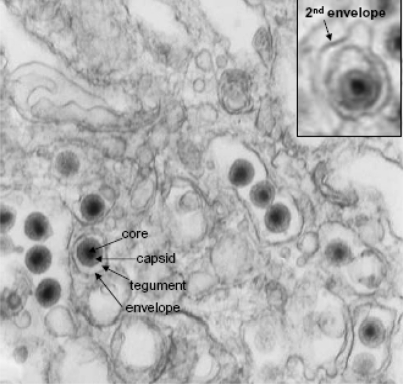
Electron micrographs of CyHV-3 infected CCB cells showing the ultrastructure of mature virions in the cytoplasm. Top right insert shows a secondary envelopment of a mature virion at the periphery of the infected cytoplasm.

When evaluated by this section electron microscopy, uninfected CCB cells have an oval shaped nucleus, with chromatin displaying slight clumping (heterochromatin), and a prominent nucleolus. The nuclear envelope is thin and densely stained. The cytoplasm contains slender, elongated mitochondria, located mostly in the zone directly adjoining the nucleus (Figure 3A). The nucleus of CyHV3-infected CCB cells (Figure 3B) has two distinct zones: a central, electron lucent area, and a marginal zone of condensed dark matter, showing accumulation of chromatin and small particles which appear to be capsid precursors. Morphologically, CyHV3 virus particles are typical of herpesviruses (Figure 4). It has a nonsymmetrical electron-dense core that contains the viral genome. This is surrounded by a 100 to 110-nm-diameter icosahedral-shaped capsid embedded in a tegument with thread-like structures and encased in a envelope [17,94–96]. In studies on assembly in infected cells these nucleocapsids appear to bud out of the inner nuclear membrane to a perinuclear space then lose this primary envelope as they cross the outer nuclear membrane into the cytoplasm. The virus acquires a secondary envelope as they bud into cytoplasmic vesicles or membrane folds in the peripheral zone of the infected cell [96]. This process is similar to that seen in *Herpesviridae* [95].

### 4.2. Molecular Characteristics of CyHV3

Pulse field gel electrophoresis (PFGE) results [98], later confirmed by publication of the full genomic sequence [3], revealed that CyHV3 bears a 295 kbp genome which is larger than any known *Herpesvirales* member [60]. The large genome may be a characteristic of the CyHV’s as PFGE revealed similar genomic size of CyHV1 to that of CyHV3 [99]. CyHV2 is difficult to grow in tissue culture and is lost after several passages *in vitro* [16,99]. The inability to isolate CyHV2 in large quantity impedes the assessment of its genomic size and structure.

#### Expression:

This large genome encodes 156 predicted genes (open reading frames-ORFs) [3]. Each of CyHV3 ORFs is transcribed into at least a single mRNA. The viral transcriptome can be classified into Immediate Early, Early and Late genes [100]. However, the number of proteins actually expressed by the viral transcriptome is unknown. Analysis of the CyHV3 structural proteins by liquid chromatography tandem mass spectrometry identified 40 structural proteins comprising 3 capsid, 13 envelope, 2 tegument, and 22 unclassified proteins [74]. These include the type 3 membrane protein expressed by ORF 81, which is located on the viral membrane and is probably the most immunogenic protein [101].

Like several other herpesviruses, CyHV3 bears genes which encode for genes related to factors of the immune system such as interleukin-10 (IL-10, ORF 134), lipoprotein (ORF 68), tumor necrosis factor receptor (TNFR-1, ORF 4L) and TNFR-2 (ORF 12). It remains to be determined the role that these genes play to enhance virus-survival in its natural hosts. However, the IL-10, ORF 134 and TNFR-1 and ORF 4L genes are nonessential for virus multiplication in cultured cells. Table 3 shows the viral genes that are known to be non-essential for virus propagation in cultured cells [102]. This information is critical for developing efficient vaccines.

### 4.3. CyHV3 Disease Management

The devastating nature of the disease on the Koi and Carp industry has led to several strategies to control the spread of the virus. Improved diagnostic methods, employing PCR and ELISA, brought about measures such as stamping out, disinfection and control of fish movements to be put in place in countries importing and producing carp and koi.

#### Resistant strains:

One way to reduce the threat caused by CyHV3 is to select strains and crossbreeds which are more resistant to viral infection. Screening of several edible carp strains and their crossbreeds revealed that the Dor-70 × wild-type Sassan fish were quite resistant to CyHV3 infection (60.7% survivors) [103]. However, the design and development of carp strains resistant to CyHV3 will require the use of modern molecular genetic methodologies such as quantitative trait loci and microarrays [104]. Recently an association between polymorphism in the MHC class II gene of common carp and resistance to CyHV3 was shown [105]. Genotyping analysis and identification of SNP markers within the common carp innate immune genes can be employed to identify genetic linkages to resistant strains [106]. Even so, the breeding system may not be appropriate for selecting resistant ornamental koi fish. Immunization of fish against the virus may be a useful tool to overcome the CyHV3 threat. Unfortunately, thus far, efforts to immunize carps with inactivated virus or with viral proteins have proven unsuccessful. To eradicate this disease from fish husbandries, two methods of fish immunization were developed in Israel: immunize of the fish with the pathogenic virus (natural immunization) or with an attenuated CyHV3.

#### Natural immunization:

Based on the observation that the disease breaks out when the water temperature is between 18 °C and 28 °C, Dr. I. Bejerano, Central Fish Health Laboratory, Ministry of Agriculture and Rural Development, Israel, developed a protocol for selecting carp and koi with naturally acquired immunity. According to this procedure, healthy fingerlings were exposed to the virus by cohabitation with sick fish for 2 to 5 days at 22 °C to 24 °C (permissive temperature). Thereafter, the water temperature was elevated above 30 °C for 25 to 30 days, and the fish were then transferred to open-air ponds. This procedure was found to be quite efficient, and 60% of the immunized fingerlings survived a challenge with sick fish [77]. Fish surviving the procedure are immunized against a challenge infection for years post exposure [107]. Although this procedure was beneficial to Israeli carp fisheries, it has several disadvantages: (i) by using this method, farmers spread the pathogenic virus over many fisheries and risk spreading it into wild carp populations; (ii) the procedure involves a loss of 40% or more of the fingerlings; (iii) economically the procedure is costly (iv) because the pathogenic CyHV3 used for immunization may persist in the fish body and could reproduce following stress, inducing the disease in the infected fish themselves and/or in non-immunized fish and (v) by employing this method the pathogenic virus is perpetuated.

#### Attenuated virus:

Live, attenuated vaccines have many advantages in aquaculture [108]. In general, live vaccine stimulates all phases of the immune system, resulting in balanced systemic and local responses involving both humoral and cellular branches of the immune system. The advantages of using a live attenuated virus vaccine are especially prominent in fish, where heat-inactivated virus is poorly immunogenic and large amounts of proteins are required for achieving an efficient and durable immune response [109,110]. However, the chance that reverted mutated virus will appear and threaten immunized populations is very small. Experiments to achieve a nonpathogenic attenuated virus have been carried out in Israel since 2003 [103,111]. The attenuated virus was isolated following serial transfer of the Israeli CyHV3 isolate in KFC. Viruses harvested after 20 passages in culture induced the disease in a small percentage of naïve fingerlings following injection or bathing [103,111]. It can be postulated, therefore, that the genetic alterations that accumulated in both the viral and host cell genomes facilitated the isolation of an attenuated virus. The attenuated virus was cloned in tissue culture in order to avoid undesired recombination, complementation, and reversion to a pathogenic virus. “Back passage” studies, in which the virus strain was extracted from vaccinated fish shortly after vaccination and used to reinfect naïve fish, were performed in an attempt to select for a reverting pathogenic virus. A five time serial passage *in vivo* showed no reversion of the attenuated strain to the pathogenic wild type phenotype [112].

Several cloned viruses were UV irradiated and then re-cloned in order to insert additional mutations into the viral genome [113–115]. Currently, the selected attenuated virus clone does not induce the lethal disease and efficiently protects the immunized fish against challenge infection [77,111]. Carp are very sensitive to pathogenic and attenuated viruses, and a short immersion of fish in water containing virus is sufficient for infection. The infection of fish with pathogenic and attenuated viruses is temperature restricted; fish held at the nonpermissive temperature immediately following infection were not affected by the pathogenic virus and were not rendered resistant to the disease. The attenuated virus must propagate in the host fish in order to induce intensive protection against the virus. Like the pathogenic virus, which induces the disease only at the permissive temperature, the attenuated virus requires the appropriate temperature to confer protection. Efficient protection is achieved by immersing the fish in water containing the attenuated virus for 40–60 min, followed by incubation at the permissive temperature for an additional 48 to 72 h [111]. Protection against CyHV3 is associated with elevation of specific antibodies against the virus. The CyHV3-specific antibody titer rises after 7 d.p.i. and peaks at 21 d.p.i. [77]. The levels of anti-CyHV3 antibodies remained high in fish injected with either the pathogenic or the attenuated virus for a period of 50–60 days, after which there is an apparent gradual decline in antibody titer [116]. Although levels of free anti-CyHV3 antibodies were low in fish tested at 280 d.p.i, 100% of the immunized fish were protected for a challenged infection, suggesting a presence of specific efficient memory cells. The modified live attenuated vaccine is produced by Kovax Ltd. (Jerusalem, Israel) and, since 2005, is widely used as a preventive measure in koi and carp farms in Israel.

#### Length of immunity:

Field observations in Israel of both vaccinated and “naturally immunized” fish suggest a prolonged protection against a wild type infection employing either method. Yet these observations may be influenced by the endemic presence of wild type CyHV3 in ponds, eliciting a re-infection and thereby prolonging the presence of antibodies. To determine the duration of time fish are protected, Perelberg and co-workers [116] conducted a controlled study in which vaccinated fish were maintained in a virus free environment. Samples were collected periodically and challenged with wt CyHV3. The study reports a high level of protection maintained over a period of 280 days post vaccination. Virus inoculation and then maintenance of fish at 14 °C, 24 °C and 31 °C induced comparable anti-virus protection, whereas the level of the antibody titer was about 4–5 times lower at the lowest temperature compared with that at the high temperature. A practical consequence deduced from these results is that fish kept at a wide range of temperatures can be efficiently immunized, provided they are also maintained at permissive temperatures for a certain time after inoculation.

### 4.4. Latency

#### “Latency” in tissue culture:

A distinctive biological property of the alloherpesviruses is their dependence on thermal conditions. The fact that their respective hosts adapt to a wide temperature range required their viruses to do the same. Similar to CyHV3, CyHV1 replicates at temperatures ranging from 10 °C–25 °C but not at 30 °C [117]. CyHV3 temperature dependence is intrinsic to its replication and transcription both *in vivo* and *in vitro*. Cell culture studies using common carp brain cells (CCB) reveal that the permissive temperature range in which disease presents itself in fish correlates to viral replication, transcription and CPE induced in a cell culture model [98]. The transfer of CyHV3 infected CCB cells to 30 °C results in viral replication arrest coupled with a down regulation of viral mRNA transcription. Prolonged incubation of cells under non-permissive conditions revealed that CyHV3 persistence in cultured cells may be limited. Infected cells showed recrudesce of viral replication and onset of CPE following 30 days at 30 °C but not following 70 days at the elevated temperature conditions. The conditional quiescence in which CyHV3 persists in poikilothermic vertebrate host cells maintained under non-permissive conditions enables it to survive within the host without killing it, giving the virus an evolutionary advantage. It is plausible that this genetic characteristic of the poikilothermic vertebrate viruses could be developed into genes responsible for latency in large DNA viruses of homoeothermic vertebrate.

#### Latency in fish: High *versus* low temperature:

Mammalian and avian herpesviruses are highly adapted to their hosts, and lethal infection is usually observed only in fetuses, in immunosuppressed organisms, or following infection of an alternative host. Herpesviruses establish lifelong latent infection, a feature which is the hallmark of all herpesviruses [118]. In contrast, infection with CyHV3 causes an acute disease with greater than 90% mortality of both juvenile and adult carp. Assuming CyHV3 to be “truly” a herpesvirus suggests that it undergoes a latent infection in its host. Recent studies suggest a unique form of latency in infected carp. Real time PCR detected CyHV3 at 64 d.p.i in fish that had survived primary infection, exhibiting no clinical signs [92]. Gilad *et al.* [92] showed that infected fish kept at low temperatures (13 °C) following infection, exhibit no onset of disease and are healthy in appearance. When maintained at the low temperature for 30 days and then transferred to 23 °C the fish developed disease, and mortality rates were eventually as high as controls maintained at the permissive temperatures throughout the trial. Yet no mortality was observed in an infected group maintained for a 64 day period at 13 °C and then transferred to 23 °C. In contrast, evidence of a viral reactivation 30 weeks after initial exposure and maintenance at 12 °C was shown by St-Hilaire *et al.* [119]. Fish infected and maintained at low temperatures were exposed to a temperature stress, which induces the disease, again suggesting temperature dependence for viral latency [63,119]. More recent publications [63,120] show the presence and possible latency of CyHV3 in leukocytes of fish that had been exposed to the virus. Latency and persistence studies demonstrate that a relatively low percentage of the infected population “carries” the virus [63,119]. Reactivation of the disease is apparently linked to maintaining the population at a low temperature and then shifting the temperature to the permissive range [63].

## 5. Ictalurid Herpesvirus 1 — A Model Clade 2 Herpesvirus

Ictalurid herpesvirus 1, the first intensively studied of the alloherpesviruses, was first characterized in 1971 [20]. This virus was first isolated from populations of juvenile channel catfish in Alabama, Arkansas and Kentucky suffering massive mortalities due to a severe hemorrhagic disease termed channel catfish virus disease (CCVD) in 1968 [19]. IcHV1 is the best characterized *Alloherpesvirdae* Clade 2 and is in many respects on the opposite end of the spectrum of *Alloherpesviridae* when compared to CyHV3. As we review IcHV1 we will compare it to CyHV3 to illustrate alloherpesvirus conservation and diversity.

### 5.1. IcHV3 — Disease Overview

CCVD affected populations of fish may experience high mortality, reduced growth and a predisposition to bacterial diseases [121]. The severity of a CCVD epizootic is significantly enhanced by environmental stress and crowding. Losses on operations vary substantially from year-to-year without obvious environmental cues, changes in management or genetics of the stock. IcHV1 is thought to be maintained in a population by vertical transmission. IcHV1 specific PCR on recently hatched fry from 5 representative fingerling operations demonstrated latent carrier status in 10–20 percent in each population of the sampled fish [122]. Given that all five populations were endemic and these farms represented approximately 20% of the commercial catfish fingerling production it can be assumed that IcHV1 is endemic in most aquaculture populations of channel catfish in the Southeastern United States.

#### Channel catfish virus disease:

Outbreaks of CCVD are sporadic in heavily stocked channel catfish fingerling ponds. Although older fish can be affected [123], natural outbreaks almost exclusively occur in young-of-the-year channel catfish during the warm summer months. The optimum temperature for disease progression is 27 °C or higher [124]. In the catfish producing region of United States, these temperatures occur from July through September when fingerlings are less than 4 months of age. Under these conditions over 90% of the population may die in less than 2 weeks from the first signs of disease, yet most IcHV1 endemic populations will experience no obvious CCVD and no substantial mortality.

#### Clinical Signs:

During a CCVD outbreak IcHV1 is highly contagious and spreads quickly though the population. Clinical signs appear two-three days post infection and include erratic swimming, exophthalmia, a distended abdomen and hemorrhages in the fins. Internal gross pathology includes yellow ascites, swollen spleen and posterior kidney.

#### Histopathology:

In contrast to CyHV3 infections where gill, and skin are important sites of pathology, the most significant early histological changes occurring with IcHV1 infection are extensive edema, inflammation and necrosis of renal hematopoietic tissue and tubules. This is followed by focal necrosis, hemorrhage and edema of the liver and gastrointestinal tract and necrosis of pancreatic tissue, congested spleen and focal areas of hemorrhage in musculature [125,126].

#### Virus isolation:

IcHV1 can be readily cultured in brown bullhead (BB) or channel catfish ovary (CCO) cell lines [127]. IcHV1 causes syncytia that contract, forming raised clumps with radiating cytoplasmic spindles. This CPE can be first seen after 12 hours at 30 °C and spreads rapidly. Complete involvement of the cell monolayer and lifting from the flask surface can occur within 12 hours of the first sign of CPE.

#### Host specificity:

Like other alloherpesviruses IcHV1 is very host specific. Natural outbreaks of CCVD have only been reported in channel catfish, the closely related blue catfish (*Ictalurus furcatus*) and channel × blue catfish hybrids. Experimental challenges of yellow bullhead *Ameiurus natalis*, brown bullhead *A. nebulosus* and black bullhead *A. melas* catfish, European wels catfish *Silurus glanis* African (*Clarias gariepinus*) and Asian catfish (*Clarias batrachus*) were resistant to infection [128–130]. Whereas blue catfish were moderately susceptible and channel × catfish hybrids were as susceptible as channel catfish [131]. IcHV1 is also very host-specific in cell culture. Other than CCO and BB cells, it has been found to replicate in *Clarias* kidney cell line K1K. We have evaluated the growth characteristics of a virus isolated from blue catfish. This isolate has a broader cell host range than previously characterized IcHV1. Notably the blue catfish isolate replicated in chinook salmon embryo cell line (CHSE-214). When CHSE-214 cells were infected with CCVLacZ (a β-galactosidase expressing construct of IcHV1), many cells stained blue with X-gal indicating that the restriction was not in penetrating the cell. Partial sequencing of the DNA polymerase of the blue catfish virus demonstrates that it is a IcHV1 strain. The 500 bp sequence revealed 97% nucleotide identity and 100% amino acid identity to the type isolate [57].

#### Dissemination of the Virus:

Using radio labeled IcHV1 in immersion challenges Nusbaum and Grizzle demonstrated likely virus uptake in the gills and caudal fin of the fish followed by a build up in the liver, gallbladder and gut [132]. Cell culture titrations and competitive quantitative PCR preformed on immersion exposed catfish fingerlings demonstrate highest virus titers occurred in the posterior kidneys with peak virus production occurring 3–4 days post infection and this correlated well with the peak mortality period [133]. The next highest concentration of virus occurred in the gills. Analysis of water demonstrated detectable shedding on days 2–5 post infection with day 4 being the peak [133].

### 5.2. Molecular Characteristics of IcHV1

Early electron microscopic analysis, sucrose density gradient sedimentation, reassociation kinetics, and restriction fragment assays on the IcHV1 genome demonstrated that the mature package genome is linear, non-permuted ∼130 kb with 18Kb direct repeats [134,135]; there are genetic differences between strains [136] and that the virus loses one copy of the direct repeats and becomes endless in infected cells (concatemeric or circular) [137]. In 1992 Davison sequenced 134,226 bp linear genome of IcHV1 and predicted that it contained 79 ORFs encoding 76 genes (the DNA polymerase gene-ORFs 57, 58, and terminase gene ORFs 62, 69 and 71 are spliced) [2]. This was the first alloherpesvirus sequenced and the striking lack of homology led Davison to conclude that IcHV1 was extremely distantly related to mammalian herpesviruses. This genome is less than half of the size and encodes less than half of the genes of CyHV3. The purified IcHV1 genome was also shown to be infectious and efficiently undergo homologous recombination allowing for trait and mutation analysis by marker rescue/marker transfer [138]. These studies and, more recently, the ability to efficiently generate recombinants in *Escherichia coli* using overlapping genomic fragments cloned into bacterial artificial chromosomes has open the way for efficiently evaluate IcHV1 genes by reverse genetics [139].

In 1995, Davison and Davison using proteomic analysis of differentially fractionated preparations of purified virions, demonstrated that 11 of the predicted genes encode structural proteins, one on the envelope (gene 59-major glycoprotein), two tegument proteins (genes 11, 65), four tegument associated proteins (genes 15,72,73,74) and four capsid proteins (genes 28, 27, 39, 53) [140]. The quantitative data of the capsid proteins was used in conjunction with cryoelectron microscopy and three dimensional image reconstruction by Booy *et al.* in 1996 to deduce the detailed morphology and make-up of the capsid [61]. They demonstrated that the structure of the IcHV1 capsid was remarkably similar to that of herpes simplex virus 1. Other characterized gene products include the identification of the gene 50 product as a secreted mucin [141,142] and the thymidine kinase product of gene 5 which can activate nucleotide analogs including acyclovir [143]. The thymidine kinase gene is likely derived from host deoxycytidine kinase gene [144]. More recently Kunec *et al.* [145] used high-throughput mass spectroscopy with probabilistic proteogenomic mapping to identify expression of 37 of the predicted genes and identify the products of 17 novel protein coding regions.

In 1980, Dixon and Farber evaluated the temporal profile of virus protein expression in IcHV1 infected cells and found that the genes are temporally regulated much like the more intensely studied members of *Herpesviridae.* Of 32 identified infected cell proteins, 8 were expressed within 2 hours of infection (immediate early candidates), 8 were expressed 2–4 hours after infection (early candidates) and 16 were expressed after 4 hours post infection (late candidates). Furthermore like other herpesviruses some of the immediate early protein candidates were expressed at higher levels following release from protein synthesis inhibitor, cycloheximide, and the synthesis of late protein candidates was inhibited by Ara-C, an inhibitor of DNA synthesis [134]. Similar temporal expression assays were performed on various transcripts coded by the direct repeat regions. However, transcription analysis has been problematic in that many genes utilize the same termination/polyadenylation signals resulting in overlapping transcription [146,147]. Also, when trying to differentiate immediate early genes from early genes using cycloheximide blocking, some early genes are apparently leaky, and these early gene transcripts can build up to high levels over time [148,149]. The reason for “leaky” early gene transcription is not known, but among the 14 genes in the direct repeats genes 1 and 3 are clearly immediate early genes [149].

### 5.3. Molecular Analyses of Host Pathogen Interaction

Herpesviruses and their hosts have intricate interactions at both the cellular and organism level. The precise processes have not been worked out for IcHV1, but it is known that upon infecting the cell the virus rapidly suppresses host protein synthesis [134]. Also the virus causes reorganization of the cytoskeleton [150]. There is a redistribution of actin filaments and a loss of organization of microtubules and vimentin filaments associated with syncytia formatin. Disruption of microtubules with nocodazole inhibits cell fusion and virus production. The addition of taxol (a microtubule stabilizer) to the nocodazole treated cells rescues virus production, showing its dependence on microtubules [150].

The cell type also appears to influence the interaction. Chinchar *et al.* evaluated IcHV1 replication in a macrophage cell line, a B lymphocyte cell line, a T-lymphocyte cell line and BB cells and found that all lines were susceptible [151]. The B cells were most permissive, and showed rapid CPE characterized as cell ballooning, while T cells and macrophages showed delayed replication and reduced virus yields and CPE characterized as disaggregation of natural cell clusters and lysis. When protein expression was evaluated, B cells displayed virus gene expression and a shutdown of host protein synthesis, whereas the macrophage and T cells displayed no shutdown of host gene expression and a 10 hour delay of virus gene expression [151]. More recently, the gene expression of IcHV1 infected CCO cells and B cells were compared using microarray analysis and differential expression of interferon response genes (ISGs) were noted [152]. Both lines up-regulated interferon alpha, ISG-15, IRF-4, and STAT-1, but CCO cells up-regulated Mx, while B cells down-regulated Mx and up-regulated IRF-1. These differences may help explain the rapid death observed in B cells when compared to the other susceptible cells. Mx protein is an important inhibitor of virus replication and had been previously shown to be up regulated in catfish in response to IcHV1 and Poly I:C and the earlier induced expression of Mx in Poly I:C treated fish was associated with better survival [153]. Also, pre-exposure of CCO cells to catfish reovirus provides protection to IcHV1 and this protection was associated with proteins released into the media (likely interferons) [154]. Anti-viral activity was also naturally expressed in un-infected T-cell and macrophage cultures but not B-cell cultures, possibly explaining the differential sensitivity [154].

We lack details on the roles innate and acquired defenses play in IcHV1 resistance. Naïve channel catfish have been shown to have a cell population that kills IcHV1 infected cells and this appears to be analogous to the mammalian natural killer cells [155]. Catfish are efficiently immunized CCVD infection using attenuated vaccines and these exposed fish as well as survivors of natural infections develop neutralizing antibodies [155–157] as well and antibodies to numerous non-neutralizing epitopes [157,158]. These IcHV1 specific antibodies may play an important role in protection; as passive transfer of neutralizing sera from adult catfish to juvenile provides protection [159]. Also, IcHV1 antibodies can persist for over 2 years after exposure [123,160]. The antibody levels apparently increase during summer months [160], and may be boosted by periodic virus recrudescence [157]. Furthermore, early-life-stage resistance was correlated to neutralizing antibodies in the maternal parent suggesting that maternal transfer of antibodies may play a role in preventing disease outbreaks [161].

### 5.4. Latency and Vertical Transmission

Since early studies on CCVD, latency and vertical transmission had been suspected. This was based on the presence of IcHV1 neutralizing titers in the brood fish that had produced CCVD affected fry in one of the first documented outbreaks [156]. Also, IcHV1 antigen (but no culturable virus) was detected in ovarian tissue of immunosuppressed adult fish immediately after spawning [162]. In 1985, Bowser *et al.* isolated IcHV1 from adult catfish during the winter when temperatures were below 8 °C and this isolation was enhanced if the fish were immunosuppressed with dexamethazone and the leukocytes co-cultured with CCO cells [163]. Then in 1985 Wise *et al.* reported the detection of IcHV1 DNA in asymptomatic adult channel catfish using Southern blot analysis [164]. Later this method was used to demonstrate that offspring from IcHV1 carriers were positive for the IcHV1 genome but were negative for infectious virus [165]. Subsequently several IcHV1 specific PCR assays were developed and used to evaluate carrier fish [65,166,167]. The presence of a latent virus in naïve, susceptible offspring presents an interesting dilemma. What prevents virus expression and disease? We have found the carrier rate within from positive spawns are 40–75% of the fish [161] and these carrier fry are susceptible to CCVD when exposed to exogenous IcHV1 [133,161]. Since the isolation of virus from adult carriers in the winter, no other research has reported successful induction of IcHV1 recrudescence. In a recent study when fish were experimentally infected and held for two months then induction from recrudescence was attempted using dexamethazone and leukocyte co-cultivation, no virus was isolated [157]. However, sporadic virus gene expression occurred and a boosting of the antibody levels occurred, suggesting partial virus expression. These data suggest that, like CyHV3, temperature influences virus re-expression.

### 5.5. CCVD Management

Most commercial channel catfish fingerling producers reduce their losses to CCVD by avoiding overly stressing and crowding fingerlings during the hot periods and by keeping stock densities moderate. In some regions producers try to avoid carrier brood stock by evaluating the fish for IcHV1 specific antibodies or by using PCR to detect latent virus.

Several experimental CCVD vaccines have been developed. These include a DNA vaccine [168], an attenuated live virus vaccine produced by multiple passages of IcHV1 in *Clarias* cells [169], a thymidine kinase gene deleted recombinant attenuated virus [170] and a gene 50 deleted recombinant virus [171]. The DNA vaccines appear equivocal in effectiveness against CCVD [172] where as the attenuated live vaccines are very effective. One advantage of the recombinant vaccines is they can express foreign genes and thus can function as a vaccine vector- providing protection against both CCVD and another pathogen [173]. The infectious bacterial artificial chromosome system for IcHV1 in conjunction with an *in vitro* recombination system greatly facilitates vaccine vector construction and optimizing foreign gene expression [174].

## 6. Conclusions

Alloherpesviruses represent an important group of pathogens affecting fish. They are very divergent from members of *Herpesviridae* but have similar biological properties and host pathogen relationships. These traits were acquired through a common distant ancestor and/or by parallel evolution. CyHV3 and IcHV1 are the two most characterized of the alloherpesviruses. They are model species representing the two distinct clades of alloherpesviruses that infect fish. Examination of these two pathogens shows that they share basic replication traits, they both establish latency in their host and temperature is an important factor in regulating host-pathogen interactions. Intensification of aquaculture and global trade of live fish will likely bring to light many new, challenging diseases caused by alloherpesviruses. Hopefully, the powerful new molecular methods will allow us to better understand how these pathogens function, spread and cause disease so we can better control them.

## Figures and Tables

**Table 1.** A summary of molecular characteristics, disease characteristics and cell culture of amphibians (based on [10]).

**Virus name (abbreviation)**	**Family, Clade ^1^ (*Genus*)**	**Common name (abbreviation)**	**Host(s)**	**Disease**	**Cell line ^3^- CPE, temperature**	**Ref.**
Anguillid HV 1 (AngHV1)	Alloherpesviridae, 1	HV anguillae (HVA)	Japanese eel *Anguilla japonica* and European eel *A. Anguilla*	Hemorrhages of skin, fins, gills, liver	EK, EO-1, EP-1, BF-2, FHM, RTG-2, 20–25 °C syncytia and rounded cells	[4,11]
Cyprinid HV 1 (CyHV1)	Alloherpesviridae, 1 (*Cyprinivirus*)	HV cyprini, carp pox HV, carp HV(CHV)	Common carp *Cyprinus carpio*	High losses in fry- exopthalmia hemorrhages, survivors have papillomas	KF-1, EPC, FHM 15–20 °C. Cells rounded and vacuolated	[12,13]
Cyprinid HV 2 (CyHV2)	Alloherpesviridae, 1 (*Cyprinivirus*)	Goldfish hematopoietic necrosis virus (GFHNV)	Goldfish *Carassius auratus*	High mortality all ages. Necrosis of hematopoietic tissue, spleen, pancreas, intestine	GF-1 (EPC, FHM)-unreliable, characterized by PCR and sequencing	[14–16]
Cyprinid HV 3 (CyHV3)	Alloherpesviridae, 1 (*Cyprinivirus*)	Koi HV (KHV), carp nephritis and gill necrosis virus (CNGV)	Common carp	gill inflammation, hyperplasia, and necrosis, hematopoietic tissue necrosis, high mortality,18–26 °C, all ages	KF-1, CCB, CFC, Au, Tol/FL Vacuolation after 4 days at 20 °C.	[17,18]
Ictalurid HV 1 (IcHV1)	Alloherpesviridae, 2 (*Ictalurivirus*)	channel catfish virus (CCV), Channel catfish herpesvirus	Channel catfish *Ictalurus punctatus*	Kidney, liver and intestinal necrosis, hemorrhages, high mortality in young fish at above 27 °C	CCO, BB 30 °C, syncytia	[19,20]
Ictalurid HV 2 (IcHV2)	Alloherpesviridae, 2 (*Ictalurivirus*)	Ictalurus melas HV (IcmHV)	Black bullhead *Ameiurus melas*	Kidney necrosis, hemorrhages, high mortality all ages	CCO, BF-2	[21,22]
Acipenserid HV 1 (AciHV1)	Alloherpesviridae, 2	White sturgeon HV 1	White sturgeon *Acipenser transmontanus*	diffuse dermatitis, high losses in juveniles	WSSK-1 syncytia 15 °C	[23]
Acipenserid HV 2 (AciHV2)	Alloherpesviridae, 2 (*Ictalurivirus*)	White sturgeon HV 2	White sturgeon	Epithelial hyperplasia	WSSK-1, WSS-2 rounded vacuolated cells 15 °C	[24]
Salmonid HV 1(SalHV1)	Alloherpesviridae, 2 (*Salmonivirus*)	HV salmonis (HPV) Steelhead herpesvirus (SHV)	Rainbow trout *Oncorhynchus mykiss*	Mild disease low losses at 10 °C. Adults- Virus shedding in ovarian fluid. No signs of disease.	RTG-2, CHSE 214, 10–15 °C extensive syncytia	[25]
Salmonid HV 2(SalHV2)	Alloherpesviridae, 2 (*Salmonivirus*)	Oncorhynchus masou virus (OMV)	Cherry salmon *O. masou*, coho salmon *O. kisutch*, sockeye salmon *O. nerka*, coho salmon *O. keta*, rainbow trout,	Viremia, external hemorrhages expthalmia, hepatic necrosis with high losses in young. Survivorsoral papillomas, virus shed in ovaran fluid	RTG, CHSE 214,15 °C syncytia	[26,27]
Salmonid HV 3 (SalHV3)	Alloherpesviridae, 2 (*Salmonivirus*)	Epizootic epitheliotropic disease virus (EEDV)	Lake trout *Salvelinus namaycush*, lake trout × brook trout *S. fontinalis* hybrids	Epithelial hyperplasia, hypertrophy, hemorrhages on eye and jaw. High losses in juveniles at 6–15 °C	EM, PCR and sequencing	[28,29]
*Gadid herpesvirus* 1 (GaHV1)	Alloherpesvirdae, 2	Atlantic cod herpesvirus (ACHV)	Atlantic cod *Gadus morhua*	Hypertophy of cells in gills. High losses in adults.	EM, PCR and sequencing	[30]
Ranid HV 1 (RaHV1)	Alloherpesviridae, 2 ^2^ (*Batrachovirus*)	Lucké tumor HV (LTHV)	Leopard frog *Rana pipiens*	Renal adenocarcinoma	EM, tumor explant culture	[31]
Ranid HV 2 (RaHV2)	Alloherpesviridae, 2 ^2^ (*Batrachovirus*)	Frog virus 4 (FV-4)	Leopard frog	No known disease	ICR-2A	[32,33]
Pilchard HV	Alloherpesviridae, 2		Australian pilchard *Sardinops sagax*	Acute losses with gill inflammation, epithelial hyperplasia and hypertrophy	EM, PCR and sequencing	[34–37]
tilapia HV	Possible Herpesviridae	Tilapia larvae encephalitis virus (TLEV)	Blue tilapia (*Oreochromis aureus*)	Encephalitis and high loses in larvae	EM, PCR and Sequencing	[38]
Percid HV 1 (PeHV1)		HV vitreum, walleye HV	Walleye *Stizostedion vitreum*	diffuse epidermal hyperplasia	WO, WC-1, We-2. syncytia, 4–15 °C.	[39]

1 Clades designated by Waltzek *et al.* [10];

2 Ranid herpesviruses suggested to be in a separate clade (subfamily) [40];

3 Cell names are: Au—goldfish fin, BB—brown bullhead, CCB—common carp brain, CCO—channel catfish ovary, CHSE 214—Chinook salmon embryo, EK-1—eel kidney, EP-1—eel epidermis, EPC—carp papilloma (cell lines now of fathead minnow origin [41]), FHM—fathead minnow, GF—goldfish fin, ICR—leopard frog embryo, KF-1—koi fin, CFC—carp fin, RTG-2—rainbow trout gonad, Tol/FL—silver carp fin, WC—walleye fibroblast, We-2—walleye embryo, WO—walleye ovary, WSS-2—white sturgeon spleen, WSSK-1—white sturgeon skin.

**Table 2.** Probable fish herpesviruses detected by electron microscopy.

**Virus name (abbreviation), Common name (abbreviation)**	**Host(s)**	**Disease**	**Ref.**
Esocid HV 1 (EsHV1), Pike epidermal proliferative HV, pike HV	Northern pike *Esox lucius* and muskellunge *E. masquinongy*	blue spot disease-Flat, granular, bluish-white skin lesions caused by enlarged epidermal cells	[42]
Pleuronectid HV 1 (PlHV1), HV scopthalami	Turbot *Scopthalamus maximus*	Giant cells (polykaryocytes), greatly enlarged cells in skin and gill epithelium	[43,44]
Flounder HV (FHV)	Japanese flounder *Paralichthys olivaceous*	Epidermal hyperplasia, epidermal cells with virus particles, high losses of fry	[34]
Golden ide HV	Golden ide *Leuciscus ide*	Epidermal hyperplasia, papillomas-referred to as carp pox	[45]
Pacific cod HV	Pacific cod *Gadus macrocephalus*	Hypertrophy of epidermal cells	[46,47]
Sheatfish HV (SHV)	Wels catfish, *Silurus glanis*	Epidermal hyperplasia, papillomas	[48]
European Smelt HV, Smelt papillomatous virus, HV of Osmerus eperlanus	European smelt *Osmerus eperlanus*	Papillomas and Hyperplastic skin lesions on dorsal fin- virions are comet shaped	[49,50]
Rainbow smelt HV	Rainbow smelt *Osmerus mordax*	Papillomas and squamous cell carcinomas	[51]
Smooth dogfish HV	Smooth dogfish *Mustelus canis*	Epidermal depigmented lesionsepidermal cell necrosis with virus particles	[52]
Atlantic salmon HV	Atlantic salmon *Salmo salar*	Papillomas especially on smolts	[53]
Angelfish HV	Angelfish *Pterophyllum altum*	Skin hemorrhages, swollen spleen and liver, virus seen in splenic macrophages	[54]
Red striped rockfish HV	Red striped rockfish *Sebastes proriger*	Hepatomegally, Giant cells (polykaryocytes), hemorrhage, necrosis and inflammation in liver	[55]

**Table 3.** Non-essential genes of CyHV3.

**Gene/ORF**	**Function**	**Class**	**Location**
ORF 52	Unknown	E	Unique large
ORF 105	Unknown	E	Unique large
ORF 55	TK gene	E	Unique large
ORF 139	B22RH gene	E	Unique large
ORF1	hypothetical protein	IE	Terminal repeat
ORF 3L	TF gene	IE	Terminal repeat
ORF6L	hypothetical protein	IE	Terminal repeat
ORF134	IL-10 like gene	E	Unique large
ORF 4L	TNFR-1 like gene	E	Unique large
ORF 141*	RNR gene	E	Unique large
ORF 16 **	GPCR gene	E	Unique large

* Controversial: seems to be essential;

** deleted by Costes *et al.* [97]; E: early gene; IE: immediate early; TK: thymidine kinase; TF: transcription factor; IL-10: interleukin 10; TNFR: tumor necrosis factor; RNR: ribonucleotide reductase (large subunit); GPCR: G protein coupled receptor gene.
